# Human preferences for cognitive and emotional capabilities in robots across different application domains

**DOI:** 10.3389/frobt.2025.1511549

**Published:** 2025-03-26

**Authors:** Hilda Nääs, Sam Thellman, Tom Ziemke

**Affiliations:** Department of Computer and Information Science, Linköping University, Linköping, Sweden

**Keywords:** human-robot interaction (HRI), social robots, cognitive robotics, user preferences, human-centered robotics, robot design, robot ethics

## Abstract

People’s preferences regarding cognitive and emotional capabilities in robots need to be considered in the design of robotic systems that align with human values and expectations. This study investigates how such preferences vary across different robotics application domains and identifies key influencing factors. In a between-subjects study with 271 participants, both quantitative and qualitative data were collected on preferences for 12 mental (cognitive and emotional) capabilities in six types of robots, each situated in a specific domain: healthcare, defense, household, social, education, or customer service. The results reveal a general preference for agency-related abilities (e.g., planning, reasoning) over experience-related abilities (e.g., feeling happiness, pain) across all domains. However, there was a weaker preference for agency capability in household cleaning robots and a stronger preference for experience capability in social companionship robots. Qualitative analysis revealed a common desire for robots to function objectively and logically, without emotions, while still showing empathy toward human mental states. Additionally, gender and educational background emerged as factors influencing participants’ preferences. Unlike previous research, which mainly focused on the attribution of mental capabilities to robots, this study offers insights into human preferences and the factors shaping them, which can inform the design of future robots and help facilitate their successful integration into society.

## 1 Introduction

In navigating social situations, humans strongly rely on the perception or attribution of mental states—such as beliefs, desires, intentions and feelings—to others, based on inferences drawn from observable behavior (Leslie, 2001). A substantial body of human‐robot interaction (HRI) research indicates that such *folk‐psychological* interpretations are not limited to human and animal behavior but are also highly prevalent in people’s interpretations of the behavior of robotic systems (Thellman et al., 2022). This has been argued to be due to the fact that the human mind tries to understand and explain robot behavior based on the same conceptual framework normally used to explain human behavior (de Graaf and Malle, 2019).

While both causes and effects of mental state attribution to robots are well studied (e.g., Butler et al., 2019; Cucciniello et al., 2023; de Graaf and Malle, 2019; Gray and Wegner, 2012; Saltik et al., 2021; Thellman et al., 2023; Wykowska, 2024; Zhao and Malle, 2022), relatively little is known concerning what preferences people hold regarding mental capabilities in robots. A pioneering study on such robot mind preferences by Malle and Magar (2017) found that people generally wanted logical robots without emotions, but still able to have empathy. The study found no differences in preferences across domains (domestic, nursing, military). Thellman et al. (2023) conducted a survey exploring people’s preferences for higher-order mental states in artificial agents, such as robots’ beliefs about human intentions. Their findings indicate that these preferences vary based on the agent’s context and function, with participants favoring specific higher-order mental states in certain types of artificial agents. Building onto this work, we aim to deepen the understanding of people’s preferences regarding mental capabilities in robots by adopting a more expansive approach. This includes exploring a broader range of robot application domains, investigating human factors influencing these preferences, and collecting qualitative data regarding participants’ reasoning and thoughts, all while relying on an existing two-dimensional model of mind (Gray et al., 2007; McMurtrie, 2023).

As robots become increasingly integrated into various domains of society, understanding these preferences is important for facilitating successful human-robot interactions across different contexts. People might, for example, want their own social companion robot to have certain emotional capabilities, and they might want automated vehicles to understand the goals and intentions of vulnerable road users, but they might not be willing to let robotic sales staff read their mind and influence their shopping decisions. Hence, the objective of this study is to investigate if preferences for mental capacities in robots vary across different application domains and identify potential factors influencing these preferences. While previous research mainly has focused on mind perception/attribution in interactions with robots, this study shifts the focus to individuals’ preferences, aiming to fill a knowledge gap in the HRI literature. The following research questions guided the investigation:

• RQ1: Do participants’ preferences for capabilities of agency and experience in robots vary across different application domains?
• RQ2: Do participants’ demographics (age, gender, educational background) affect preferences for capabilities of agency and experience in robots?

## 2 Methods

The study employed a between-subject design (*N* = 271), collecting both quantitative and qualitative data through six surveys, each focusing on a robot type situated in a specific application domain. An *a priori* G^*^Power analysis suggesting 216 participants for One-Way ANOVA guided the sample size. Participants were recruited through convenience sampling at Linköping University’s campuses in Linköping and Norrköping (Sweden), and via online distribution of a survey link. Inclusion criterion was being over 18 years old. This recruitment process resulted in a culturally homogeneous sample of Swedish-speaking participants. The sample included 115 (42.4%) women, 155 (57.2%) men, one (0.4%) participant identified as “other” (excluded from gender analyses). Age ranged from 19 to 76 years (*M* = 26.3 years, *SD* = 10.2), with 208 (76.8%) aged 18–25 years. Educational background included 17 (6.3%) participants without university or college education, 160 (59.0%) participants with an ongoing or completed Bachelor’s degree, and 94 (34.7%) participants with an ongoing or completed Master’s degree. Computer science education was reported by 77 (28.4%) participants (51 (66.2%) men and 26 (33.8%) women). The remaining 194 (71.6%) participants had no such education.

There are numerous domains in which robots can be useful and beneficial, from both a professional and domestic standpoint. However, due to feasibility reasons the study was limited to include six different application domains to assess RQ1. The selection was guided by an aim to encompass a wide spectrum of contexts in society: healthcare, defense, household, social, education, and customer service. Participants were randomly assigned to one application domain and informed about the robot type operating within the domain and a description of its specific purpose. This information is detailed in Table 1.

**TABLE 1:** The different robotics application domains investigated in the study, including robot types and descriptions of how they operate.

Application domain	Robot type	Description
Healthcare	Surgical Robot	A medical robot designed to perform surgical procedures on patients
Defense	Military robot	A military robot designed to perform and participate in military operations
Household	Cleaning robot	A cleaning robot designed to perform cleaning tasks and maintenance in indoor environments
Social	Companion robot	A social robot designed to act as a friend and provide companionship in everyday life
Education	Educational robot	An educational robot adapted to act as a teacher and supervisor in a teaching environment
Customer service	Service robot	A service robot designed to assist and serve customers in a restaurant environment

The dependent variable for both research questions was preferences for mental capabilities. This measure draws on Gray et al.’s (2007) seminal study of mind perception that identified two dimensions of mind: *experience* (emotion-related) and *agency* (action-related). A conceptual replication by McMurtrie (2023) refined the model, confirming its robustness, and this refined model was adopted as the guiding framework for measuring mind in the current study. It might be worth noting that we here, in line with McMurtrie, use the broad term *mental* capabilities to refer to both *cognitive* capabilities (on the agency dimension) and *emotional* capabilities (on the experience dimension). For feasibility reasons, the 22 mental capabilities encompassed in McMurtrie’s model were here reduced to twelve, six from each dimension. The selection process was guided by five criteria: (1) remaining balance across dimensions, (2) excluding capabilities requiring bodily senses (e.g., feeling hunger), (3) avoiding overly similar capabilities (e.g., “can explain their decisions” and “can provide reasons for their actions”), (4) excluding vaguely phrased capabilities (e.g., “can feel emotion”), and (5) prioritization based on strength of alignment with respective dimensions in the factor analysis. This process yielded the final set of 12 capabilities shown in Table 2.

**TABLE 2:** The 12 mental abilities included in the study, corresponding to agency and experience dimensions of mind, following McMurtrie (2023).

Experience	Agency
Can feel happy	Can plan for the future
Can feel pleasure	Can understand a person’s goals
Can feel pain	Can explain their decisions
Can feel panic	Can praise moral actions
Can love specific people	Can disapprove of immoral actions
Can have intense urges	Can reason logically

The 20-item questionnaire was created using LimeSurvey. It began with demographic questions about gender, age, educational level, and whether participants had a background in computer science, serving as independent variables for RQ2. Participants then were introduced to one robot type in a specific application domain and asked to imagine its appearance and operation, and to keep this in mind throughout the survey. Participants provided a brief description of the imagined robot in free text. The following 12 items assessed preferences for mental capabilities, formulated as: “To what extent do you want the robot you imagine to have the following mental abilities?”. Specific capabilities were presented in a randomized sequence, with responses captured on a visual analog scale (also known as “slider scale”) from 0 (no ability) to 100 (full ability). Visual analog scales produce similar responses as Likert scales and text entry formats when assessing mental states (Couper et al., 2006). While they impose slightly higher cognitive load (Couper et al., 2006), we chose them for their ability to provide more precise, high-resolution measurement (Betella and Verschure, 2016). After assessing their preferences, participants were offered the chance to add free text comments regarding their thoughts, explanations and opinions, to complement their answers. This qualitative element in the study aimed to collect data on how participants reasoned and motivated their answers. All study data are available at https://osf.io/renvt/.

## 3 Results

### 3.1 RQ1: Do participants’ preferences for capabilities of agency and experience in robots vary across different application domains?

#### 3.1.1 Agency

A One-Way ANOVA assessed the effect of application domain on agency preferences, measured by mean score for the six mental capabilities corresponding to the agency dimension of mind. The analysis revealed a statistically significant effect with a medium effect size, *F* (5, 265) = 3.50, *p* = 0.005, partial *η* ^2^ = 0.081. Post hoc Tukey tests showed statistically significant differences between the cleaning robot and all the other robot types, except for the service robot. Specifically, differences were found between:

• The surgical robot and the cleaning robot (mean difference = 15.8, *t* = 3.65, *p* = 0.004).
• The military robot and the cleaning robot (mean difference = 16.8, *t* = 3.84, *p* = 0.002).
• The companion robot and the cleaning robot (mean difference = 14.5, *t* = 3.45, *p* = 0.008).
• The educational robot and the cleaning robot (mean difference = 17.0, *t* = 4.04, *p* = 0.001).

**FIGURE 1:**
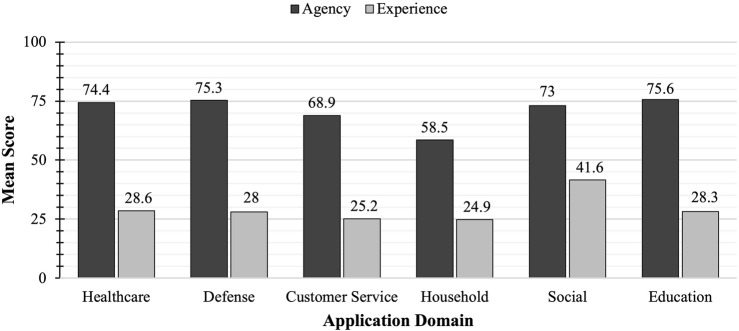
Mean scores for agency and experience preferences by robotic application domain. Application domain showed significant effect with medium effect sizes on preferences for both agency and experience capability. Post hoc tests revealed the cleaning robot (household domain) as an outlier with weaker preference for agency-related abilities and the companion robot (social domain) as an outlier with stronger preference for experience-related abilities.

The mean scores for agency across different robot types ranged from 68.9 to 75.6, where the cleaning robot with a mean score of 58.5 was identified as an outlier, indicating weaker preferences for agency capability (Figure 1). The data met assumptions for normal distribution of residuals and homogeneity of variance.

#### 3.1.2 Experience

A One-Way ANOVA assessed the effect of application domain on experience preferences, measured by mean score for the six mental capabilities corresponding to the experience dimension of mind. The analysis revealed a statistically significant effect with a medium effect size, *F* (5, 265) = 3.73, *p* = 0.004, partial *η* ^2^ = 0.068. Post hoc Tukey tests showed statistically significant differences between the companion robot and all the other robot types, except for the surgical robot. Specifically, differences were found between:

• The service robot and the companion robot (mean difference = −16.5, *t* = −3.76, *p* = 0.003).
• The cleaning robot and the companion robot (mean difference = −16.7, *t* = −3.68, *p* = 0.004).
• The educational robot and the companion robot (mean difference = −13.3, *t* = −2.99, *p* = 0.046).

Mean scores for experience across different robot types ranged from 24.9 to 28.6, where the companion robot with a mean score of 41.6 was identified as an outlier, indicating stronger preferences for experience capabilities (Figure 1). The data met assumptions for normal distribution of residuals and homogeneity of variance.

### 3.2 RQ2: Do participants’ demographics (age, gender, educational background) affect preferences for capabilities of agency and experience in robots?

#### 3.2.1 Age

A Pearson correlation test revealed no statistically significant correlation between age and preferences for agency and experience.

#### 3.2.2 Gender

An independent t-test revealed a small, but statistically significant gender difference with small effect size for experience preference, with a stronger preference in women (*M* = 33.3, *SD* = 21.7) compared to men (*M* = 27.0, *SD* = 22.3), *t* (268) = −2.33, *p* = 0.021. No statistically significant differences were found for agency preferences between women (*M* = 71.9, *SD* = 20.0) and men (*M* = 70.5, *SD* = 21.0).

#### 3.2.3 Educational background

A One-Way ANOVA assessing the effect of participants’ educational level on experience preference revealed a small statistically significant effect with small effect size, *F* (2, 268) = 3.30, *p* = 0.046, partial *η* ^2^ = 0.021). A Tukey *post hoc* test showed statistically significant differences between participants with an ongoing or finished Bachelor’s degree and participants with no university or college education (mean difference = −13.4, *t* = −2.37, *p* = 0.048). The mean experience scores for participants with education at the Bachelor’s level was 28.7, and at the Master’s level or higher was 28.8, whereas participants with no university or college education had a mean score of 42.0, indicating higher preference for experience capabilities in robots. No statistically significant effects were found for the effect of educational level on agency preference. The data met the assumptions for normal distribution of residuals and homogeneity of variance.

When investigating the effect of computer science education, an independent t-test revealed a small statistically significant difference with small effect size in experience preference. Individuals with computer science education showed a weaker preference (*M* = 24.8, *SD* = 22.1) compared to individuals without (*M* = 31.4, *SD* = 22.1), *t* (269) = −2.24, p = 0.026. No statistically significant differences were found for agency preferences between individuals with computer science education (*M* = 74.4, SD = 20.1) and without (*M* = 69.6, SD = 20.8).

### 3.3 Qualitative analysis

The survey collected qualitative data by asking all participants to provide a brief description of the appearance of the robot they imagined would be best suited for the specific application domain. Moreover, after assessing their preferences for mental capabilities, participants were given the opportunity to include free text comments expressing their thoughts, explanations and opinions in free text to complement their answers. Of the 271 participants, 90 provided free-text answers, of which six were excluded during the initial data cleansing due to irrelevant answers (e.g., answering “No, thanks.” in the text box), leaving 84 responses for further qualitative analysis. Thematic analysis was applied manually and conducted by the first author (HN), following the step-by step guide presented by Braun and Clarke (2006). Repeatedly mentioned aspects were identified, forming the themes presented below. The quotes provided here are translated from Swedish.

#### 3.3.1 Preference for absence of feelings

This theme was supported by comments from 46 participants (54.8%) that expressed a desire for a robot free of emotional capabilities. They emphasized the importance of the robot being objective and driven solely by logic, without emotional influence. Many participants expressed a preference for the robot to function purely as a tool, welcoming the ability for it to understand practical aspects of situations. A robot with emotions, consciousness or feelings was by many participants described as both unnecessary and frightening. The following statements illustrate the theme:

- *“I find it unsettling to give robots an emotional life. They stop being robots at that point.”* (P37, service robot)
- *“I think it would feel unsafe if a robot were to have consciousness or emotions.”* (P111, companion robot)
- *“[Ability to be] Satisfied also means [ability to be] dissatisfied - scary. It’s foolish [for robots] to [have ability to have] panic and feel pain. - But [it is] important [for robots] to understand situations that may arise.”* (P22, service robot)
- *“Apart from its ability to perform what it is designed to do, it doesn’t seem necessary to add more functionality.”* (P49, service robot)

#### 3.3.2 Preference for empathy

Another theme identified in seven responses (8.3%) was a preference for a robot without feelings of their own, yet capable of having empathy and understanding towards human physical- and mental states. Participants expressed a need for the robot to be able to perform empathic reasoning regarding humans’ emotional states. For instance, one participant stated: *“I think the robot should understand and reason about emotional, mental, and logical abilities, but not have feelings or any personal desires. More like an advanced personal assistant.”* (P139, companion robot). The following statements from other participants illustrate their reasoning more deeply and illustrate how the preference spans across robots in different domains:

*In terms of pain and similar experiences, I don’t expect robots to FEEL pain, but it would be nice if they could be programmed to UNDERSTAND pain, both physical and emotional, how it manifests and what consequences it might have. The above applies to most human traits that have been mentioned [in the previous quantitative assessment of mental abilities]. I neither want or expect a robot to be like a human, but if it were possible to program an understanding of human differences and variations, that would be desirable.* (P66, educational robot)

*For me, it’s not important at all that the robot can feel pain, but rather understand how the sensation of pain affects different individuals in different ways. Also, the understanding that different procedures and interventions can cause more or less pain, postoperatively, for the patient and use that as a judgment.* (P241, surgical robot)

#### 3.3.3 Sympathy for the robot

This theme emerged from responses by seven participants (8.3%) that expressed sympathy and consideration for a robot capable of having feelings. Some participants expressed unease about robots having an emotional life, explaining that a robot with such capabilities would surpass its role as a technical agent and therefore deserve the same respect as living agents. The following excerpt illustrates one participant’s reasoning about this:

*Since the robot has a service role, I believe that negative emotions become less relevant, as they do not contribute to fulfilling the purpose of service. However, I think that if the robot cannot express negative emotions, perhaps people interacting with the robot will treat it in a wrong and unjust way. With this perspective, one views the robot as partly human and deserving of decent treatment and interaction.* (P13, service robot)

Participants also reflected on the ethical implications of owning a robot that expresses emotions. One participant even drew parallels to the ownership of a slave: *“Honestly, I have no idea how much emotion you would want to give a robot since, in practice, you own it like a slave. It becomes more about ethics than functionality.”* (P182, cleaning robot).

Other participants expressed a sympathetic desire for robots with emotional capabilities to be shielded from negative emotions, allowing them to exclusively experience positive ones. This tendency is clearly expressed by the following statements: “*Regardless of whether the robot would have any capacity for emotions, I wouldn’t want it to feel bad or suffer unnecessarily*” (P188, cleaning robot), *“There’s no real point to negative emotions like panic or pain. I see no problem with positive emotions like joy and satisfaction.”* (P50, service robot).

#### 3.3.4 Moral concerns

A concern among 16 participants (19.0%) was the ability for robots to navigate morality. Many expressed the existing challenge of defining morality amongst humans. Since the assessment and reasoning about morality vary between individuals, participants questioned how a robot would be able to accurately determine which moral path to follow. Participants reasoned about how the non-objective nature of morality would make it problematic for robots to take moral stances and how it would be difficult to judge the correctness of their decisions. *“The issue of morality is difficult because it depends on what the person programming it [the robot] considers to be moral and their opinions about morality. And who says that person is right? It becomes a very strange and complex issue.”* (P261, surgical robot).

This concern is also accounted for in the following statement:

*The robot must have some kind of morality that can stop people who are trying to use the robot to harm others. Therefore, I believe the robot must have some understanding of what is right or wrong. But this is difficult - what is right and wrong?* (P76, educational robot)

Additionally, participants raised the issue that agents capable of making moral decisions must be held accountable and take responsibility for the outcomes of their actions. One participant stated: *“‘Individuals’ who cannot be held accountable should not be able to make moral decisions or similar judgments.”* (P86, educational robot).

## 4 Discussion

Key findings indicate a general preference for robots with high agency capability and low experience capability across all application domains. These results are also reflected in the qualitative analysis, which showed that participants expressed a desire for objective and logical robots functioning without emotions. The results support the findings of the pioneering study by Malle and Magar (2017) that identified a similar pattern of individuals’ desire for robots to have objective reasoning while being free of affective capabilities. Although emotional robots appear undesirable, both studies nevertheless found a desire for robots with capability for empathy and understanding of human mental states. This tendency can appear somewhat contradictory when considering empathy as an emotional capability. However, in relation to the two-dimensional model of mind (McMurtrie, 2023), it could be interpreted as an agency-related ability associated with morality, reasoning and understanding of others’ goals and minds. To determine how the specific capability for empathic reasoning should be treated, a dimensionality reduction addressing this as a distinct ability is necessary and suggested for future research, as individuals appear to express significant preferences for this unique ability.

Unlike the findings of Malle and Magar (2017), this study found application domain to be an influential factor for preferences of robot’s mental capabilities. Specifically, the cleaning robot emerged as an outlier with weaker agency preference, while the companion robot had stronger experience preference than the others. An interpretation of the preference in the companion robot is found in the qualitative analysis, which revealed that participants described their imagined appearance of the robot using terms such as cute, sweet, and small, and likened it to the role of a pet. This characterization of the robot as harmless and non-threatening combined with its role as a social companion might have encouraged people to anthropomorphize it. By attributing human-like traits such as emotional connection and care, this may have contributed to the preference for experience capabilities. This interpretation aligns with findings by Gray et al. (2007), who found a correlation between attributing experience-related abilities to an agent and a tendency to avoid causing harm to it. Thus, the preference may reflect a broader inclination to treat agents perceived as benign or friendly with greater empathy and consideration.

While no specific explanation for the weaker agency preference in the cleaning robot is found in the qualitative analysis, findings by Thellman et al. (2023) may offer valuable context guiding an interpretation. Their study found stronger preference for Theory of Mind abilities (understanding of others’ mental states, strongly connected to agency-related abilities such as understanding of others’ goals) in self-driving cars compared to virtual agents. The authors speculated that this preference might stem from the physical embodiment of self-driving cars giving the consequences of its actions potential life-or-death outcomes. The low agency preference in the cleaning robot could similarly be related to a perceived irrelevance of its ability to take responsibility for its actions, as it operates in less critical scenarios compared to, for example, the military robot or the surgical robot. This interpretation aligns with findings by Gray et al. (2007), as it describes a correlation between agents attributed with high agency capability being judged to have more responsibility for their own actions. Given this, future research should further explore the hypothesis that perceived responsibility is a driving factor behind preference for agency capabilities in robots, particularly in domains where the consequences of their actions carry greater moral weight.

Our findings further suggest that demographic properties could influence preferences for mental capabilities in robots. Both gender and educational background showed small effects on preference for experience capabilities. Although the limited scope of this study did not allow for a deeper exploration of these tendencies, the results may prompt further research in this area. Age did not show significant impact on preferences. However, the skewed distribution of age in the dataset could have compromised the quality of the analysis and further research exploring the variable more comprehensively would be desirable.

Beyond addressing the research questions, the results shed light on ethical considerations crucial for the responsible development of robots. Participants expressed discomfort with the scenario of owning a robot with feelings or consciousness. Moreover, participants questioned the feasibility of robots navigating moral dilemmas, given the subjective nature of morality and the variability in human assessments of moral correctness. Concerns regarding the possibility of holding moral decision-making agents accountable for their actions was also expressed by participants, where they emphasized the need for mechanisms to ensure their responsibility and liability in accordance with their decisions. This nuanced perspective underscores the complex ethical considerations involved in the integration of robots into society. Although robots are not likely to *have* actual feelings or minds, our findings suggest that it might be advisable to avoid designing robots *expressing* such abilities. A central finding of this study is that expression of cognitive and emotional capabilities in robots should be tailored to their specific application domains, which means that designers stand to benefit from being mindful ensuring that technological advancements not only follow the direction of technical innovation, but also resonate with human preferences.

## Data Availability

All study data are available at https://osf.io/renvt/.
